# Acute lower respiratory tract infections and associated factors among under-five children visiting Wolaita Sodo University Teaching and Referral Hospital, Wolaita Sodo, Ethiopia

**DOI:** 10.1186/s12887-021-02888-6

**Published:** 2021-09-20

**Authors:** Birhanu Wondimeneh Demissie, Esayas Aydiko Amele, Yibeltal Asmamaw Yitayew, Zemen Mengesha Yalew

**Affiliations:** 1 School of Nursing, College of Health Science and Medicine, Wolaita Sodo University, Sodo, Ethiopia; 2 Department of Paediatrics and Child Health Nursing, College of Medicine and Health Science, Wollo University, Dessie, Ethiopia; 3 Department of Comprehensive Nursing, College of Medicine and Health Science, Wollo University, Dessie, Ethiopia

**Keywords:** Acute, Respiratory tract infection, Associated factors, Under-five, Ethiopia

## Abstract

**Background:**

Lower respiratory infections are a leading cause of morbidity and mortality worldwide, particularly in children younger than 5 years. Even if the burden of lower respiratory infections in children under 5 years old had decreased dramatically in the last 10 years, it is still the main cause of morbidity and mortality in children under-5 years old in developing countries, so the aim of this study was to assess the magnitude of lower respiratory tract infections and associated factors among under-five children visiting Wolaita Sodo University Teaching and Referral Hospital.

**Method:**

A cross-sectional study was conducted from 1st to 30th April 2019, among under-five child/mother or caretaker pairs visiting Wolaita Sodo University Teaching and Referral Hospital. Child/mother or caretaker pairs who visits outpatient department for curative care service or follow up were recruited for the study. Data were collected using a semi-structured pre-tested interviewer-guided questionnaire. Epi-info (version 7.1.2.0) was used for data entry, and Statistical Package for Social Sciences version 20 was used for analysis. Bivariate and multivariate logistic regression, crude and adjusted odds ratios with their 95 % confidence intervals was computed. Finally, a p-value ≤ 0.05 was used to identify variables that had a significant association with acute lower respiratory infection.

**Result:**

A total of 414 child/mother or caretaker pairs were recruited for the study. The magnitude of acute lower respiratory infections among under-five children was 40.3 % (95 % CI: 35.7- 44.9 %). Unvaccinated children (AOR: 2, 95 % CI, (1.27–3.16)), non-exclusive/replacement feeding (AOR: 1.85, 95 % CI, (1.18–2.91)), households mainly used unclean fuel for cooking (AOR: 2.12, 95 % CI, (1.07–4.19)), absence of separate kitchen (AOR: 1.7, 95 % CI, (1.09–2.65)), and absence of window in the kitchen room (AOR: 1.69, 95 % CI, (1.07–2.68)) showed significant association with acute lower respiratory infection.

**Conclusions:**

The magnitude of acute lower respiratory tract infections among under-five children visiting outpatient department was 40.3 %. Unvaccinated children, non-exclusive/replacement feeding, using unclean fuel for cooking, absence of a separate kitchen, and absence of window in the kitchen showed significant association with acute lower respiratory infection. Therefore, special attention should be given to the environmental sanitation and family health components of health extension packages.

## Background

Acute lower respiratory tract infections (LRTIs) are any infections in the lungs or below the voice box, which include pneumonia, bronchitis, and bronchiolitis [1]. To date, pneumonia is the most common type of lower respiratory tract infection (LRTI) and globally, 150 million new episodes of pneumonia are identified per year worldwide, more than 90 % of which occur in developing countries [2, 3]. Viruses are the most common cause of pneumonia in infants and young children, and the most frequent symptoms and signs are coughs, increased respiratory rate, fever, breathing difficulty, runny nose, and indrawn chest wall in more severe disease [3–6].

Lower respiratory tract infections (LRTIs) are the leading cause of morbidity and mortality worldwide, particularly in children younger than five years [7, 8]. Globally, LRTIs cause 704 000 deaths, and the highest under-five LRTI mortalities were in sub-Saharan Africa. Even if the burden of LRTIs in children under-five years of age has decreased dramatically in the last ten years, it is still the main cause of death in developing countries [8–10]. Each year, more than 2 million children under-five years of age die due to pneumonia in developing world, and 43 % of global under-five deaths from acute LRTIs occur in India, Nigeria, Democratic Republic Congo and Ethiopia  [11]. In Ethiopia in 2015, 25,970 under-five children died due to LRTIs, and 14,148.3 were caused by pneumonia  [8].

Different factors were identified for the increased risk of LRTIs in children. Of these, poverty, restricted family income, low parental education level, low birth weight, malnutrition, lack of breastfeeding, maternal literacy, smoking, cow dung use for fuel, low socio-demographic status, solid fuels for cooking and heating, immune impaired populations, improved toilet facilities, season, and residence [8, 10, 12, 13]. Similarly, the risk of death from LRTIs will be determined by very severe pneumonia, age below two months, diagnosis of Pneumocystis Carinii, chronic underlying diseases including HIV/AIDS, severe malnutrition, young maternal age, low maternal education, low socioeconomic status, second-hand smoke exposure, and indoor air pollution  [14].

Widespread immunization against influenza, measles, bacilli Calmette-Guerin (BCG), and now pneumococcus have been related to the decline of LRTIs in children  [3]. Likewise, vaccines prevent an estimated 2.5 million deaths among under-five children every year. However, one child dies every 20 s from a disease that could have been prevented by a vaccine  [15]. Ethiopian children suffer four to eight episodes of acute LRTIs on average every year, with the highest occurrence in urban areas of overcrowded living conditions [7]. In Ethiopia, only 39 % received all vaccinations at some time, and 22 % were vaccinated by the appropriate age  [16], this low immunization coverage will result in an increased risk of LRTI morbidity and mortality.

Even if Ethiopia has achieved millennium development goals (MDG) 4 by 2013, LRTIs are one of the most common causes of under-five children mortality  [17]. To achieve the sustainable development goal (SDG) 3 of ending preventable deaths of new-borns and under-five children by 2030, efforts to reduce deaths due to lower respiratory tract infection should remain a top priority  [18, 19]. Therefore, this study aimed to assess the magnitude of acute lower respiratory tract infections and associated factors among under-five children visiting Wolaita Sodo University Teaching and Referral Hospital (WSUTRH), 2019.

## Methods

### Study design, area and period

An institution-based cross-sectional study was conducted from 1st to 30th April 2019, at Wolaita Sodo University Teaching and Referral Hospital (WSUTRH). Wolaita Sodo University Teaching and Referral Hospital is found in Sodo town, which is located 157 km from the regional capital city, Hawassa, and 327 km from the capital city of Ethiopia, Addis Ababa. According to the Wolaita Zone Health Department information 2019, the estimated population of Wolaita Zone is approximately 2,020,386, of which approximately 983,991 are males and 1,030,396 are females. The hospital gives service to more than 3 million people in the catchment area of wolaita and Dawro, has 350 inpatient beds, and 450–500 patients visit the hospital per day [20].

### Populations

#### Source population

All under-five child/mother or caretaker pairs visiting WSUTRH pediatric outpatient department (OPD) for curative care service or follow up.

### Study population

Randomly selected under-five child/mother or caretaker pairs visited WSUTRH pediatric outpatient department (OPD) from 1st to 30th April 2019.

### Sample size determination

The sample size was calculated using a single population proportion formula and since there was no proportion of LRTI with similar population we calculate with the assumption of 50 % under-five prevalence of acute LRTIs to obtain the largest sample size, with a 95 % confidence interval and 5 % marginal error.

By considering a 10 % non-response rate, the final sample size was 422 child/mother pairs.

### Sampling procedure

The estimated number of child/mother or care taker pairs visited under-five OPDs in the study period was 1050 based on the last six months of patient flow and then k was calculated, k = 1050/422 ~ 2, participants were selected using a systematic random sampling technique (k = 2), that is every two client (child/mother pair) visiting under five OPD until the required sample size was obtained.

### Data collection instrument

A face-to-face interview was conducted using a structured questionnaire that was adopted and modified from previous studies  [21–27]. The questionnaire comprises sociodemographic factors, maternal and child factors, environmental factors, and the outcome variable acute LRTIs. Length board (< 2 years) or portable stadiometer (≥ 2 years) and portable digital weight scale were used to measure the height and weight of the child, respectively.

### Data collection procedure and quality control

The data were collected by four BSc nurses and supervised by two MSc pediatrics and child health nurse professionals. Training was given for data collectors and supervisors for two days, and pre-test was conducted in 5 % [21] of the final sample size in Sodo Christian hospital. Both supervisors and data collectors were closely followed for the data collection process, and all filled questionnaires were checked every day, and errors were corrected accordingly. The weight of the child was measured using a well-calibrated, portable digital weight scale without shoes and wearing light clothes. Moreover, reliability of the weight scale was checked before each measurement.

### Data entry and analysis

The collected data were checked for completeness, consistency, and accuracy, entered into EPI data version 3.1 and exported to Statistical Package for Social Sciences version (SPSS) 22.0 for data analysis. Descriptive statistics with percentages, frequency distributions, measures of central tendency, and dispersion were used to describe the data. Bivariate logistic regression was used to check variables having an association with ALRIs, and those variables found to have a p-value of < 0.2 were further analyzed using multiple logistic regressions. Odds ratios (ORs) with 95 % CIs were computed and variables with a p-value < 0.05 were considered significant variables. Model fitness was checked with the Hosmer and Lemeshow goodness-of-fit test, which was p = 0.767.

### Operational definitions

#### Acute lower respiratory tract infections

 A child presented with cough, fever, rapid or difficulty breathing in the last two weeks and was diagnosed with acute LRTIs (pneumonia or acute bronchitis or bronchiolitis) by the physician.

**Acute nutritional status of the child;** was assessed based on weight-for-height Z- score which was calculated with WHO AnthroPlus software.

**Sever acute malnutrition;** those who had WHO Z score of < -3 SD.

**Moderate acute malnutrition;** those who had WHO Z score of -3 to < -2 SD.

**Mild acute malnutrition/normal;** those who had WHO Z score of ≥ -2 SD.

### Main cooking fuel

***Unclean fuel:****using wood, animal dung, charcoal, crop wastes, kerosene stove and kerosene lamp* [28].

***Clean fuel:****use of electricity, gas, ethanol, and solar *[28].

### Breast feeding

***Exclusive breast feeding:*** *infants receive only breast milk for the first six months or receive only breast milk until the time of assessment for infants < six months.*

***Non-exclusive or replacement feeding:*** *Giving food or fluid (milk) alone or in addition to breast milk for infants < six months.*

### Immunization status

***Immunized for age:****Took all vaccines appropriate for age based on the immunization schedule*.

***Incomplete:****defaulters or those who were not vaccinated based on the immunization schedule*.

***Not vaccinated:****a child who did not take the vaccine at all*.

## Result

### Sociodemographic characteristics

A total of 414 child/mother or caretaker pairs had participated in the study, which resulted in a 98.1 % response rate. The mean age and standard deviation of children and mothers were 21.3 ± 14.13 months and 29 ± 5.8 years, respectively. Nearly half (46.4 %) of children were aged ≥ 24 months, the majority (54.6 %) of children were male, and 59.4 % were urban dwellers. Nearly half of the respondents (49 %) were protestant in religion, 32.6 % were unable to read and write, and 57.7 % were housewives in occupation. Nearly all (93 %) children’s parents lived together, and the average monthly income of the family was 2356 ± 1316 Ethiopian birr (Table 1).

**Table 1:** Sociodemographic characteristics of acute lower respiratory infections among under-five children/mother or caretaker pair attended at WSUTRH, 2019 (*n* = 414)

Variable ***N*** = 414	Frequency (N)	Percent (%)
Age of the child		
2–5 months	44	10.6%
6–11 months	77	18.6%
12–23 months	101	24.4%
24–59 months	192	46.4%
Sex of the child		
Male	226	54.6%
Female	188	45.4%
Age of the mother		
≤24	85	20.5%
25–34	264	63.8%
≥35	65	15.7%
Residence		
Urban	246	59.4%
Rural	168	40.65
Religion of the respondent		
Protestant	203	49%
Orthodox	122	29.5%
Catholic	46	11.1%
Muslim	43	10.4%
Educational status of mother		
Unable to read and write	135	32.6%
Able to write and read	130	31.4%
Primary school	79	19.1%
Secondary school	41	9.9%
Diploma and above	29	7%
Occupation of the mother		
House wife	293	57.7%
Government employee	31	7.5%
Private business	33	8%
Student	3	0.7%
Farmer	29	7%
Daily labor	79	19.1%
Does parents live together		
Yes	385	93%
No	29	7%
Family income		
≤1999 ETB	149	36
2000–3499 ETB	189	45.7
≥3500ETB	76	18.4

### Maternal and child factors

The mean and standard deviation of children’s weight and height were 10.2 ± 2.9 kg and 78.4 ± 12.4 cm, respectively. The majority (58.2 %) of children did not have a history of upper respiratory tract infection (URTI), and 34.1 % of children did not take immunization at all. Over two-thirds of children (70.5 %) were exclusively breastfed in the first six months, 29 % of children took vitamin A in the last months, and 4.1 % of children had severe wasting. Only 3.4 % of children had parents with chronic illness, 59.4 % of mothers took iron folate, 64.5 % of mothers took extra food during pregnancy, and 4.1 % of mothers had sexually transmitted infections (STIs) during pregnancy (Table 2).

**Table 2:** Maternal and child factors of acute lower respiratory infections among under-five children attended at WSUTRH, 2019 (n = 414)

Variable N = 414	Frequency (N)	Percent (%)
History of URTI within 2 weeks		
Yes	173	41.8%
No	241	58.2%
Immunization		
Immunized	232	56%
Incomplete	41	9.9%
Not vaccinated	141	34.1%
BF in the first 6 months (current for < 6 months)		
Exclusive BF	292	70.5%
Non-exclusive/ replacement feeding	122	29.5%
Vitamin A supplementation		
Yes	120	29%
No	250	60.4%
Not eligible	44	10.6%
Nutritional status of the child		
Sever acute malnutrition	17	4.1%
Moderate acute malnutrition	30	7.2%
Mild/normal	367	88.7%
Parental chronic illness		
Yes	14	3.4%
No	400	96.6%
Maternal iron folate intake		
Yes	246	59.4%
No	168	40.6%
Take extra meal during pregnancy		
Yes	267	64.5%
No	147	35.5%
STI during pregnancy		
Yes	17	4.1%
No	397	95.9%

### Environmental factors

Half of the respondents (49.5 %) had a family size of ≤ 4 people, there was a smoker in 6 % of households, and 82.4 % of children were cared for by their mothers. The majority of households (67.4 %) had a separate kitchen, 71 % of cooking areas had windows, and 76.2 % of households used unclean fuel for cooking (Table 3).

**Table 3:** Environmental factors of acute lower respiratory infections among under-five children attended at WSUTRH, 2019 (n = 414)

Variable N = 414	Frequency (N)	Percent (%)
Family size		
≤4	205	49.5%
≥5	209	50.5%
Smoker in the house		
Yes	25	6%
No	389	94%
Who give care for the child		
Mother	341	82.4%
House maker	73	17.6%
Separate kitchen		
Yes	279	67.4%
No	135	32.6%
Cooking area(kitchen) had window		
Yes	294	71%
No	120	29%
Main household fuel type		
Unclean fuel	357	76.2%
Clean fuel	57	13.8%

### Magnitude of acute lower respiratory tract infections

The magnitude of acute LRTIs was found 40.3 % (95 % CI: 35.7 − 44.9 %).

### Factors associated with acute lower respiratory tract infections

In the bivariate analysis, parents living together, history of URTI, vaccination status, breastfeeding in the first six months, a person who cared for the child, main cooking fuel type, a smoker in the house, a separate kitchen, a window for the kitchen, and nutritional status of the child were factors that had a p-value < 0.2. Variables that had a p-value of < 0.2 in the bivariate analysis were further analysed using multivariate logistic regression. The results of this analysis showed that immunization status, breastfeeding in the first six months, main cooking fuel, separate kitchen, and window for the kitchen were significantly associated with acute LRTIs (Table 4).

**Table 4:** Factors associated with acute lower respiratory infections among under five children attended WSUTRH, 2019 (n = 414)

Variables	Acute lower respiratory infection		COR(95%CI)	AOR(95%CI)	P- value
	Yes	No			
**Parents live together**					
Yes	151 (36.5%)	234 (56.5%)	1	1	
No	16(3.9%)	13 (3.1%)	1.9(0.9–4.1)	2.22(0.99–5.01)	0.054
**History of URTI**					
Yes	81 (19.6%)	92 (22.2%)	1.59(1.07–2.36)	1.44(0.94–2.21)	0.095
No	86 (20.8%)	155 (37.4%)	1	1	
**Immunization status**					
Immunized for age	80 (19.3%)	152 (36.7%)	1	1	
In completed	16(3.9%)	25 (6%)	1.22(0.61–2.41)	1.12(0.54–2.35)	0.762
Not vaccinated	71(17.1%)	70(16.9%)	1.93(1.26–2.95)	2(1.27–3.16)*	0.003
**Breast feeding**					
Exclusive BF	104 (25.1%)	188 (45.4%)	1	1	
Non-exclusive	63 (15.2%)	59 (14.3%)	1.93(1.26–2.96)	1.85(1.18–2.91)*	0.008
**Who care for the child**					
Mother	131(31.6%)	210(50.7%)	1	1	
Home maker	36 (8.7%)	37 (8.9%)	1.56(0.94–2.59)	1.64(0.95–2.84)	0.078
**Main cooking fuel**					
Unclean fuel	153 (36.9%)	204 (49.3%)	2.3(1.22–4.36)	2.12(1.07–4.19)*	0.031
Clean fuel	14 (3.4%)	43 (10.4%)	1		
**Smoker in the house**					
Yes	14(3.4%)	11 (2.7%)	1.96(0.9–4.44)	1.81(0.75–4.33)	0.185
No	153(36.9%)	236(57%)	1	1	
**Separate kitchen**					
Yes	100 (24.2%)	179 (43.2%)	1	1	
No	67 (16.2%)	68 (16.4%)	1.76(1.16–2.68)	1.7(1.09–2.65)*	0.021
**Window for the kitchen**					
Yes	104 (25.1%)	190 (45.9%)	1		
No	63 (15.2%)	57 (13.8%)	2(1.3–3.1)	1.69(1.07–2.68)*	0.025
**Nutritional status**					
SAM	10 (2.4%)	7 (1.7%)	1.7 (0.65–4.57)	2.54(0.88–7.39)	0.087
MAM	12 (2.9%)	18 (4.3%)	1.17 (0.55-2.48)	1.15(0.5–2.61)	0.747
Mild/normal	145 (35%)	222 (53.6%)	1	1	

* = Statistically significant at *p* value < 0.05 with 95%CI

Unvaccinated children were 2 times more likely to be affected by acute LRTIs than immunized children (p = 0.003, AOR; 2, 95 % CI: 1.27–3.16). Non-exclusively/replacement feeding children were 1.85 times more likely to be affected by acute LRTIs compared with exclusively breastfeeding children (p = 0.008, AOR; 1.85, 95 % CI: 1.18–2.91). Similarly, children living in households mainly cooking with unclean fuel had 2.12 times higher odds of developing ALRIs compared with children living in houses mainly cooking with clean fuel (p = 0.031, AOR; 2.12, 95 % CI: 1.07–4.19). Additionally, the absence of a separate kitchen had 1.7 times higher odds of acute LRTIs in children than having a separate kitchen (p = 0.021, AOR; 1.7, 95 % CI: 1.09–2.65). Finally, children who lived in households that had no window in the cooking room (kitchen) were 1.69 times more likely to be affected by ALRIs compared with those children who lived in houses that had a window in the cooking room (kitchen) (p = 0.025, AOR; 1.69, 95 % CI: 1.07–2.68) (Table 4).

## Discussion

This study showed that the prevalence of acute LRTIs among children who attended WSUTRH was 40.3 % (95 % CI: 35.7 − 44.9 %). The findings of this [research are comparable with the demographic and health survey reports of Congo (39.8 %) and Gabon (38.1 %) [12]. However, this finding is higher compared with studies conducted in South West, Ethiopia (28.1 %), [22] Wondo Genet district, Ethiopia (33.5 %), [21] the overall acute LRTIs of sub-Saharan Africa (25.3 %), [12] the Rio Grande do Sul State, Brazil (23.9 %), [29] and Rwanda (5 %) [13]. The possible reason might be the difference in the study setting, difference in socioeconomic status, living conditions and the difference in operational definition to the acute LRTI(type of lower respiratory infections) included. This study was conducted in a health facility and included other acute LRTIs in addition to pneumonia. However, the above studies were conducted in a community setting or included only pneumonia, which will result in a lower prevalence.

In this study, unvaccinated children were 2 times more likely to be affected by acute LRTIs than vaccinated children. Widespread immunizations have been related to the decline of LRTIs in children [3, 30, 31]. Similar findings were reported from studies in Gamo Gofa Zone, Ethiopia, [22] Southern Ethiopia, [23] and a systematic review and meta-analysis from the UK [32]. This might be due to the preventive effect of pneumococcal vaccine against streptococcus pneumonia and pentavalent vaccine against homophiles influenza and other respiratory pathogens.

Non-exclusive breast/replacement feed children were 1.85 times more likely to be affected by acute LRTIs compared with exclusively breastfeeding children. This might be due to breast milk contains antibodies that help children fight viruses and bacteria. Additionally, children who are breastfed exclusively for the first six months had a lower risk of acute otitis media, lower respiratory infections, gastroenteritis and diarrhea, and asthma [33, 34]. Other studies conducted in Northwest Ethiopia, [24] Achefer district, Ethiopia, [35] Kersa district, Ethiopia, [25] Gamo Gofa Zone, Southwest Ethiopia, [22] Southern Ethiopia, [23] a systematic review and meta-analysis from the UK, [32] showed similar findings.

Children living in households mainly cooking with unclean fuel had 2.12 times higher odds of developing acute LRTIs compared with children living in houses mainly cooked with clean fuel. This might be due to the use of unclean fuel might produce high levels of household air pollution, including small soot particles that will penetrate deep into the lungs. Deposited particulate matter may alter airway reactivity and will affect the ability of the lungs to fight pathogens. Exposure is high among women and young children, and as a result, it doubles the risk for childhood pneumonia [36–38]. Studies conducted in Southwest Ethiopia, [22] Gondar city of Ethiopia, [26] Wolaita Sodo, Ethiopia, [27] and Pakistan [39] reported similar findings.

The absence of a separate kitchen had 1.7 times higher odds of childhood acute LRTIs compared with having a separate kitchen. This is due to cooking in the household will result in a higher level of particulate matter concentration, and young children have increased vulnerability to household air pollution due to the longer indoor stay [39]. Similar findings were observed in a study conducted in the Wondo Genet district, Ethiopia, [21] Southwest Ethiopia, [22] and Pakistan [39].

Finally, children who lived in households that had no window in the cooking room (kitchen) were 1.69 times more likely to be affected with acute LRTIs compared with those children who lived in houses that had a window in the cooking room. The kitchen without a window had limited ventilation, and it will increase the exposure of pollutants, particularly for young children who spend much of their time in the kitchen with their mother. This finding is supported by studies conducted in Wolaita Sodo, Ethiopia, [27] Wondo Genet district, Ethiopia [21], Gondar, Ethiopia, [26] and Southwest Ethiopia [22].

### Limitation of the study

This study will be subjected to recall bias and did not consider seasonal variation. Additionally, the diagnosis for acute LRTIs was made based on the physician assessment.

## Conclusions

The prevalence of acute LRTIs among under-five children attending WSUTRH was 40.3 %. Unvaccinated children, non-exclusive/replacement feeding, households mainly cooking with unclean fuel, absence of a separate kitchen, and absence of window in the kitchen room showed significant association with acute LRTIs. Therefore, strengthening the environmental sanitation (healthy home environment) and family health (child nutrition, immunization) components of the health extension packages will have a significant contribution to the reduction of under-five acute LRTIs.

## Data Availability

Data will be available upon request from the corresponding author.

## Abbreviations

AOR: Adjusted odds ratio
BSc: Bachelors of Science
COR: Crude odds ratio
LRTI: Lower respiratory tract infection
MDG: Millennium development goal
MSc: Masters of Science
OPD: Outpatient department
SDG: Sustainable development goals
SD: Standard deviations
SPSS: Statistical Package for Social Sciences
WSUTRH: Wolaita Sodo University teaching and referral hospital
